# Case report: Congenital short bowel syndrome

**DOI:** 10.4103/0971-3026.69366

**Published:** 2010-08

**Authors:** Lalitha Palle, Balaji Reddy

**Affiliations:** Department of Radiology, Focus Diagnostic Center, Punjagutta, Hyderabad, India

**Keywords:** Congenital, malabsorption, short bowel syndrome

## Abstract

Congenital short bowel syndrome (SBS) is a relatively rare condition as compared to acquired SBS. It is associated with significant mortality and morbidity. Infants usually present with failure to thrive, recurrent vomiting, and diarrhea. It is important to suspect and diagnose this condition promptly, as early initiation of parenteral nutrition or surgery, if necessary, may result in a favorable outcome. We discuss a case of an infant aged 26 days, who presented with failure to thrive, recurrent vomiting, and weight loss. A contrast study of the gastrointestinal tract revealed a short small bowel, with malrotation. The infant was started on parenteral nutrition, but succumbed shortly thereafter to severe disseminated sepsis.

## Introduction

Short bowel syndrome (SBS) may be either congenital or acquired. Acquired cases are more common, whereas congenital short bowel is a relatively rare condition. Many theories have been proposed regarding the etiology of congenital short bowel, the most common one being that it is the sequela of necrotizing enterocolitis. We present the case of an infant who had failure to thrive and recurrent vomiting, diarrhea, and weight loss from the fifth day of birth. A barium follow-through examination revealed a short length of dilated small bowel.

## Case Report

**Figure 1:**
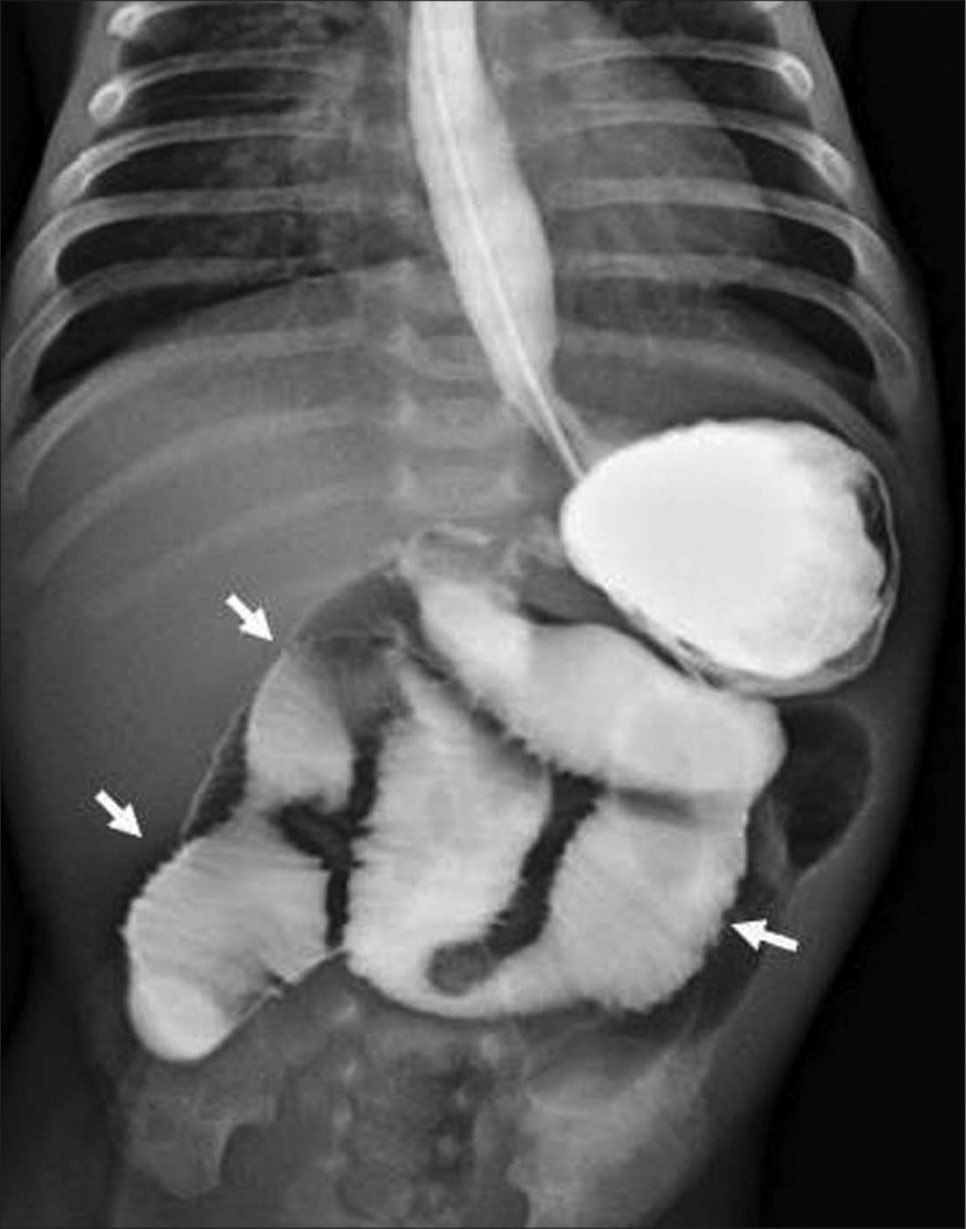
Frontal radiograph from a barium meal follow-through study shows dilated contrast-opacified small bowel (arrows); gastroesophageal reflux is seen

**Figure 2:**
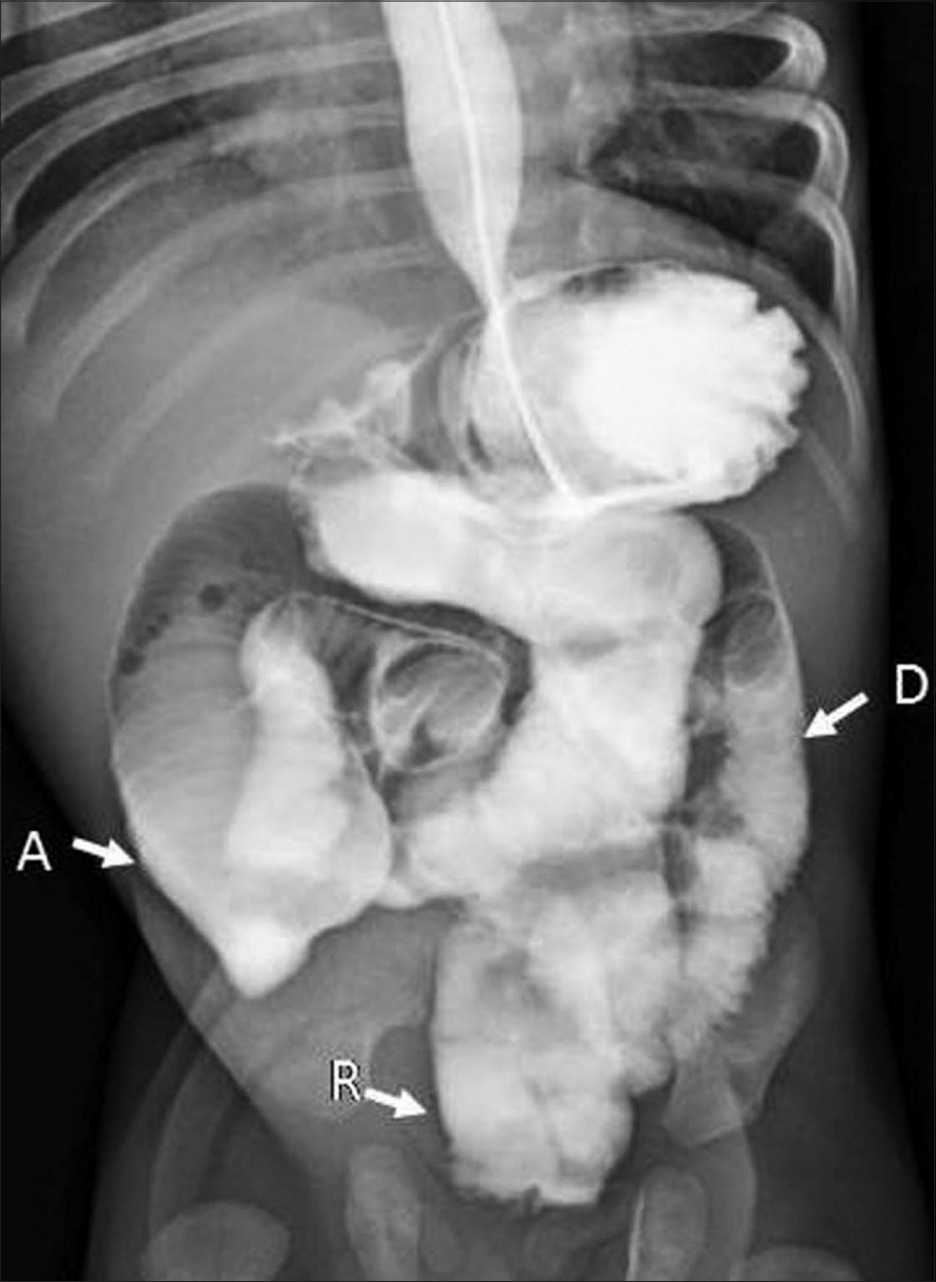
Frontal radiograph from a barium meal follow-through study shows contrast-opacified short length of dilated small bowel; A, ascending colon; D, descending colon, R, rectum. Rapid transit was noted, with contrast seen in the large bowel within 20 minutes of administration (normal mean transit time is 60 minutes)

**Figure 3:**
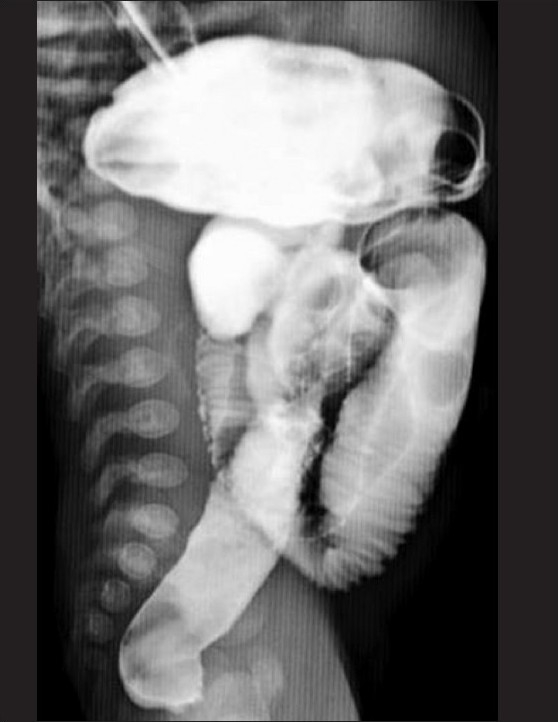
Lateral radiograph from a barium meal follow-through study shows the short length of small bowel with rapid transit (contrast is noted in the entire gastrointestinal tract, from the stomach to the rectum)

A male infant aged 26 days was brought by the parents with complaints of recurrent vomiting and diarrhea. There was a history of failure to thrive and loss of weight. The child had been born at term to healthy nonconsanguineous parents. The mother had an uneventful antenatal period, which had ended in a spontaneous vaginal delivery. The child was apparently normal at birth, with a birth weight of 3 kg. They were discharged from the hospital 2 days later, after the infant had been administered routine vaccinations. The mother complained that the baby had recurrent vomiting/regurgitation of breast milk from the fifth day of birth, with multiple episodes of diarrhea. On examination, the child appeared malnourished and weighed 2.2 kg. The abdomen was soft, with mild distention and no organomegaly. The respiratory and cardiovascular systems were within normal limits. Lab investigations revealed a hemoglobin level of 11 g%. The complete blood picture, including the platelet count, was otherwise normal. There was electrolyte imbalance, and the child was in acidosis. After correcting the electrolyte imbalance, a barium meal follow-through study was performed using diluted barium instilled via a nasogastric tube. A normal stomach and duodenal cap were seen followed by a very short length of small bowel. The entire small bowel up to the ileocecal junction measured approximately 20–25 cm. It showed dilatation with no jejunoileal differentiation [Figures 1 and 2]. Free flow of barium was noted into the large bowel and the entire colon was opacified within 20 minutes of the start of the study [Figure 3]. The child succumbed to severe disseminated sepsis shortly thereafter.

## Discussion

Congenital SBS is a relatively rare condition as compared to acquired short bowel. The first report was by Hamilton *et al*., in 1969 about the condition in two babies.[1] To the best of our knowledge, 37 cases of congenital SBS have been described in the English literature till date.[2]

The affected babies in congenital SBS are born with a short length of small bowel. Normally, the length of the small bowel in a term infant is about 250 cm.[3] There is rapid growth and elongation of the small bowel until the crown-heel length is 60 cm, after which the length of the small bowel remains relatively constant until a body length of 140 cm.[4] The signs and symptoms of SBS manifest in term infants when the length of the small bowel is less than 75 cm.[2] In the previously reported 37 cases, the shortest length of the small bowel was 20 cm, while the longest was 237 cm.

The exact cause and etiopathogenesis of this short bowel are not clearly understood. Various theories proposed include ischemia (usually secondary to necrotizing enterocoltis) causing bowel infarction,[2] defective neuroenteric development,[5] and abnormalities in the myenteric plexus.[6] SBS has been seen to be associated with other congenital abnormalities like hemivertebrae, dextrocardia, pyloric stenosis, patent ductus arteriosus, gastrochisis, meconium peritonitis, appendiceal agenesis, bowel atresia, and volvulus.[7]

The signs and symptoms of SBS manifest due to the decrease in absorptive bowel surface and because of alteration of intestinal bacterial flora. Malabsorption occurs in direct proportion to the reduction in intestinal absorptive surface area. Overgrowth of intestinal bacteria in cases of short bowel worsens this situation and causes deleterious effects in the form of inflammatory epithelial damage, leading to the release of inflammatory cytokines and mediators which disrupt the normal absorptive process.[8]

The majority of infants with SBS present with failure to thrive, vomiting, and diarrhea. Treatment can be conservative, with parenteral nutrition, or it can be in the form of surgery. Surgical treatment includes transplant and non-transplant options. Transplant options can be either intestinal or combined liver-intestinal transplantation.[9] Non-transplant options include intestinal lengthening procedures, intestinal tapering procedures for dysfunctional segments, and creation of intestinal valves.[9]

## Conclusion

SBS of congenital etiology is an uncommon disease that usually presents with failure to thrive, vomiting, and diarrhea. It is essential to recognize this disorder and to treat it accordingly.
